# Transcutaneous electrical acupoint stimulation to reduce opioid consumption in patients undergoing inguinal hernia repair: protocol for a randomized controlled trial

**DOI:** 10.1186/s13063-022-07019-9

**Published:** 2022-12-29

**Authors:** Siddarth Agrawal, Mateusz Szmit, Marek Wełna, Jerzy Rudnicki, Anil Agrawal, Waldemar Goździk

**Affiliations:** 1 Department of Internal Medicine, Occupational Diseases, Hypertension and Clinical Oncology, Wroclaw Medical University, Wroclaw, Poland; 2 Department of Pathology, Wroclaw Medical University, Wroclaw, Poland; 3 Department of General, Minimally Invasive and Endocrine Surgery, Wroclaw Medical University, Wroclaw, Poland; 4 Department of Anaesthesiology and Intensive Therapy, Wroclaw Medical University, Wroclaw, Poland; 5 Second Department of General and Oncological Surgery, Wroclaw Medical University, Borowska 213 St, 50-556 Wroclaw, Poland

**Keywords:** Complementary medicine, Pain management, Transcutaneous electrical acupoint stimulation (TEAS), Protocol

## Abstract

**Background:**

The purpose of this study is to evaluate the efficacy and safety of transcutaneous electrical acupoint stimulation (TEAS) in the postoperative treatment of patients undergoing inguinal hernia repair compared with sham and no treatment group.

**Methods:**

This study is a protocol for a three-armed, single-blinded, placebo-controlled randomized controlled trial. Ninety participants scheduled for inguinal hernia repair will be randomly assigned to the TEAS group (*n* = 30), sham group (*n* = 30), and control group (*n* = 30). The TEAS group will receive treatment using four portable coin-sized electro-stimulators at both local and distal acupuncture points. The sham group will receive sham treatment with mock electrostimulation. The treatment groups will receive mixed frequency stimulation (alternating at 2 and 100 Hz every 3 s) in continuous mode for 30 min at intervals of 2 h for 24 h postoperatively. The control group will receive postoperative pain control using patient-controlled analgesia (PCA) device. The primary outcome is the total morphine dose received in the postoperative period (mg) using PCA 24 h after surgery. The number of PCA demands (i.e., times the button will be pressed) and delivered bolus doses, score on the Visual Analogue Scale, opioid-related side effects, the requirement for supplemental medications, score on the Hospital Anxiety and Depression Scale (HADS), and blood levels of stress hormones cortisol and prolactin.

**Discussion:**

The results of this trial will determine whether TEAS with intensified stimulation protocol is a safe and effective option for reducing analgesic consumption and postoperative pain.

**Trial registration:**

ISRCTN76428396. Registered on 05 October 2020. https://www.isrctn.com/ISRCTN76428396

## Background

Patients perceive postoperative pain as one of the most unpleasant aspects of undergoing surgery. Despite numerous arguments supporting the need for effective treatment of postoperative pain, clinical reality is still far from satisfactory [1, 2]. It is reported that nearly half of all surgical patients suffer from persistent postsurgical pain [3]. Inadequate pain management may lead to severe clinical manifestations, including pneumonia, cardiovascular instability, myocardial ischemia, ileus, impaired wound healing, and anxiety [4].

Inguinal hernia repair is characterized by a high intensity of postoperative pain and patient discomfort. Severe pain is associated with tissue trauma and the placement of the preperitoneal mesh. Injury to the vulva, iliohypogastric, and ilioinguinal nerves is the most common cause of pain with a neuropathic component in the pubic and femoral areas [5]. The highest intensity of pain is recorded on the first day after surgery, and one-third of patients complain of moderate or severe pain even 7 days after the repair operation [6].

Opioids, the mainstays of most analgesia regimens during and after surgery, are responsible for dose-related side effects in 12.2% of the time, which delays recovery, incurs higher costs, leads to a longer length of stay, and increases readmission rates [7, 8].

Transcutaneous electrical acupoint stimulation (TEAS) is a non-invasive treatment modality, which combines the effects of transcutaneous electrical nerve stimulation (TENS) with acupuncture point stimulation [9]. TEAS stimulate the afferent nerves at an acupuncture point with low-voltage impulses. Most prior assessments have shown a beneficial effect of TENS or electroacupuncture (EA) in reducing pain intensity after hernia surgery [10–12]. However, to date, there are no randomized controlled trials to evaluate the efficacy of TEAS in the postoperative treatment of patients undergoing inguinal hernia repair.

In this study, for the first time, we will assess the effect of TEAS on opioids consumption in patients undergoing inguinal hernia repair. The trial is also designed to verify whether the analgesic effect of TEAS is associated with the levels of stress hormones.

## Methods and analysis

### Study objective

The primary objective is to evaluate (a) the efficacy and (b) the safety of TEAS in reducing postoperative pain after inguinal hernia repair compared with sham and standard treatment. The secondary objectives include the assessment of pain intensity (Visual Analogue Scale), frequency of complications of opiates (e.g., sedation, pruritus, nausea, vomiting), anxiety and depression (Hospital Anxiety and Depression Scale), and levels of stress hormones (blood levels of cortisol and prolactin).

### Study location

A prospective, three-armed, single-blinded, placebo-controlled randomized controlled trial will be conducted at Wroclaw University Hospital, Wroclaw, Poland. The study was planned in accordance with the Helsinki Declaration to protect the participants and was approved by the ethics committee of Wroclaw Medical University (No. KB-599/2017). The participants will.

be informed on the potential benefits, risks, alternatives, and responsibilities of the study by the researchers during the consent process.

### Study population

Participants will be recruited according to the inclusion, exclusion, and dropout criteria.

Inclusion criteria:

- Male and female patients aged 18–75 years
- Patients undergoing elective laparoscopic mesh inguinal hernia repair
- Body mass index 18–30 kg/m.^2^
- ASA classification I–III
- Patients provide signed informed consent

Exclusion criteria:

- Patients with bilateral or recurrent inguinal hernia
- Patients with a history of intolerance, hypersensitivity, or abuse of opioids
- Use of opioids in the past month
- Use of monoamine oxidase and selective serotonin reuptake inhibitors
- Patients wearing a cardiac pacemaker
- Patients with clinically significant cardiovascular, pulmonary, renal, hepatic, and neurological disease
- Patients with skin infections, surgical incision, or scar at the point of application of acupuncture
- Patients who participated in other clinical trials, or received other acupuncture therapy, in the previous 4 weeks
  Dropout criteria:
- Patients who have poor clinical compliance
- Patients who quit the RCT voluntarily will be considered as having dropped out

### Endpoints

#### Primary endpoint

Total morphine dose received in the postoperative period (mg) using patient-controlled analgesia (PCA) device.

#### Secondary endpoints

The number of PCA demands (i.e., times the button will be pressed) and delivered bolus doses after surgery will be assessed. Score on the Visual Analogue Scale prior to the surgery and at 4, 8, 12, 16, and 20 h after surgery will be recorded. The scale of 0 to 10, where 0 represents the complete absence of pain and 10 represents the worst pain intensity. Moreover, opioid-related side effects and the requirements for supplemental medications (e.g., antiemetics, antipruritics, and analgesics) will be recorded during the postoperative observation period.

The Hospital Anxiety and Depression Scale (HADS) will be used to evaluate the depression and anxiety before and at 24 h after surgery. Blood levels of stress hormones cortisol and prolactin will be measured at the same time points.

### Sequence generation

Randomization will be conducted using a computer-generated random allocation sequence using SAS 9.4 (SAS Institute Inc., Cary, NC, USA) by a statistician with no clinical involvement in this trial.

### Allocation

The allocation shall proceed via duplicate, sealed, numbered envelopes, which will be stored by the clinical trial quality control inspectors and the principal investigator. The blind bottom shall not be opened without reason during the trial. The outcome assessors and the statisticians will not participate in the treatment; they will perform the outcome evaluation and the statistical analysis independently.

### Randomization and blinding

**Fig.1:**
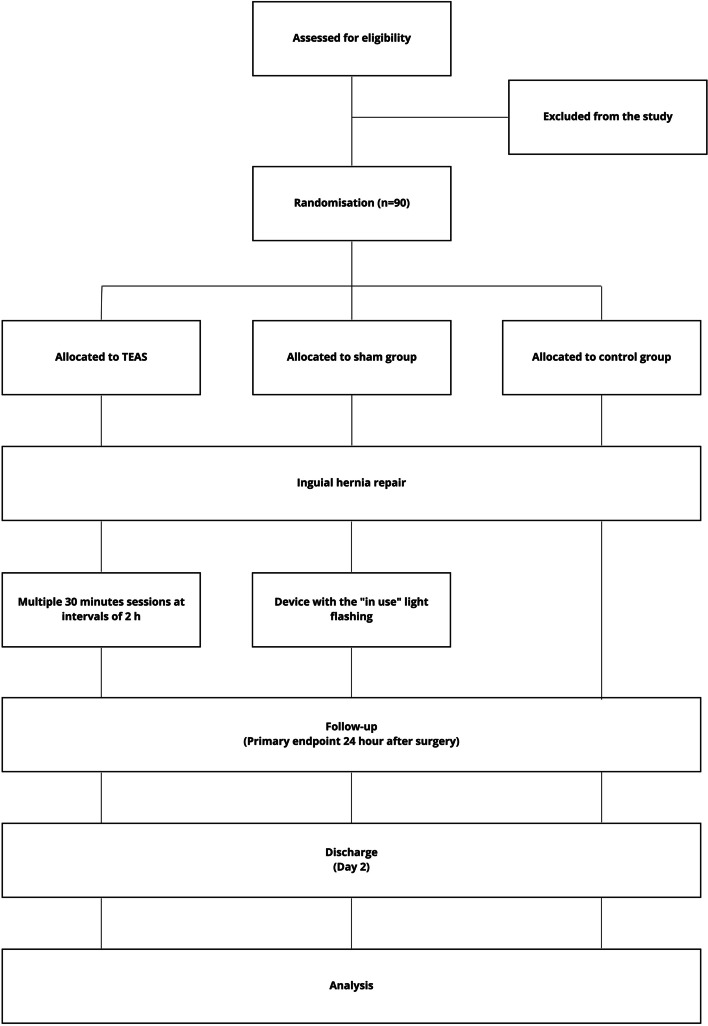
Flowchart of the trial process. TEAS, transcutaneous electrical acupoint stimulation

**Fig. 2:**
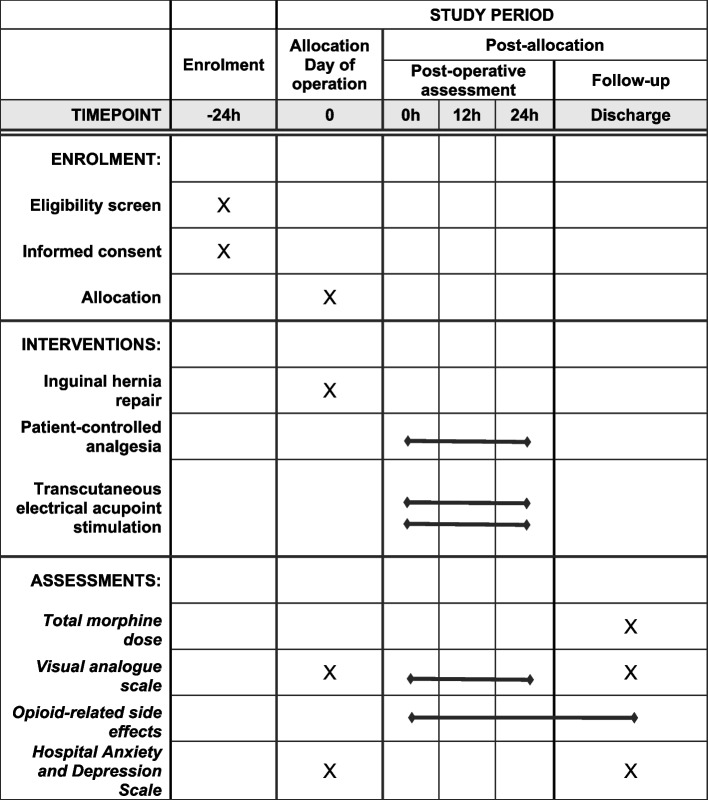
SPIRIT figure. The figure shows the phases of the trial and data collection time points

Enrolled participants will be randomly assigned to TEAS, sham, or control group (1:1:1). An independent, blinded statistician will generate the block randomization scheme. The table will be managed by an independent researcher who is not involved in the recruitment, treatment, or assessment. The participants will be blinded to the type of treatment. Mock TEAS will be provided with the same lamplight as real TEAS, so the participants will not be able to predict the allocated group based on the appearance of the treatment (Fig. 1). The practitioners will be aware of the allocation arm while the subjects, outcome assessors, and statisticians performing the data analysis will be blinded to the treatment allocation. The schedule for the study is shown in Fig. 2.

### Sample size justification

Power analysis was performed to determine the number of patients per group sufficient to.

detect a decrease of 30% or more in the PCA opioid analgesic requirements during the first 24 h after surgery, based on the results of the previous studies [13–15]. Each group will include 30 participants, a total of 90 participants. The proper size of the sample was calculated using the following formula:

$$Sample\;size=\frac{Z_{1-a/2}{^2}p\left(1-p\right)}{d^2}$$

where:

- *Z*_1_ − _*a*/2_ is the standard normal variate (at 5% type 1 error *p* < 0.05), − 1.96;
- *p* is the expected prevalence obtained from a pilot study, − 0.26; and *d* is the absolute precision, − 0.05.

### Statistical analysis

Quantitative variables will be reported using the mean and SD (M ± SD) for normally distributed data or median (Me), the lower quartile (Q1), and the upper quartile (Q3) and the range (Min–Max) for skewed data. Kolmogorov–Smirnov Normality Test will be used to verify the normality of distributions. Intergroup differences between the average values of the quantitative data will be assessed using the Student’s *t*-test (*t*-test for independents samples by groups) or Mann–Whitney *U* test. Kruskal–Wallis ANOVA will be used for multiple comparisons (post hoc tests). Additionally, a multivariate analysis, ANOVA for factorial designs (Factorial ANOVA), will be performed to analyze the main effects and their interactions (relationships between independent variables). Qualitative variables (nominal) will be represented as (*n*) and proportions (%). Independence of qualitative variables from the patient status will be verified by the chi-square test or Fisher’s exact test. The significance level will be set at 5%. All data will be analyzed using the Statistica v.13.3 (StatSoft).

## Intervention

### Anesthesia protocol

A standard premedication of 7.5-mg oral midazolam will be administered in every patient, 30 min prior to entering the operating room. Thereafter, standard patient monitoring, including continuous electrocardiography, pulse oximetry, and indirect blood pressure (measured every 5 min), will be applied and maintained until the end of anesthesia. Three-minute preoxygenation with 100% oxygen will precede the induction phase in every patient. Then, 1–3mcg/kg fentanyl, followed by 2–3 mg/kg propofol and 0.6 mg/kg rocuronium, will be applied through the peripheral vein to induce general anesthesia. Individually selected, an appropriate size endotracheal tube (ET) would be placed via direct laryngoscopy. After confirmation of proper ET placement, mechanical ventilation will be commenced and anesthesia maintained by sevoflurane 2–4 vol% mixed with 60% oxygen, titrated to achieve 0.9–1.2 MAC. Continuous monitoring of capnography will ensure proper respiratory function and gas exchange. During the surgery, additional boluses of fentanyl (1–2mcg/kg) will be administered in the presence of the patient’s reactions to pain stimuli (i.e., tachycardia/hypertension). Depending on the time of surgery, an additional bolus of 25% of the initial dose of rocuronium might be applied. For basic pain control, 1 g of metamizole will be given to every patient before completion of the surgery. After cessation of sevoflurane administration and achieving an adequate level of consciousness and responsiveness, the patient will be extubated. After the surgery, every patient will be monitored and carefully observed for at least 30 min in the recovery room.

The surgical technique will be the Lichtenstein tension-free method. Hernia patients, according to the classification of Nyhus, were type II or IIIb.

### Postoperative analgesia

On a patient’s arrival in the postanesthesia care unit, the PCA device will be connected to the patient’s intravenous line and programmed to deliver 1-ml bolus doses of morphine (1 mg) “on-demand,” with a minimal lockout interval of 10 min and a maximum 4-h dose of 15 mg according to a standardized hospital protocol. PCA therapy will be initiated in the postanesthesia care unit when the patient was sufficiently alert to understand and operate the PCA device. If the patient requires pain medication prior to starting PCA therapy, an incremental dose of metamizole 1 g intravenously, will be administered by the postanesthesia care unit nursing staff. The postoperative PCA analgesic therapy will be supplemented with TEAS/sham therapy, which will start when the patient arrives in the postanesthesia care unit.

**Fig. 3:**
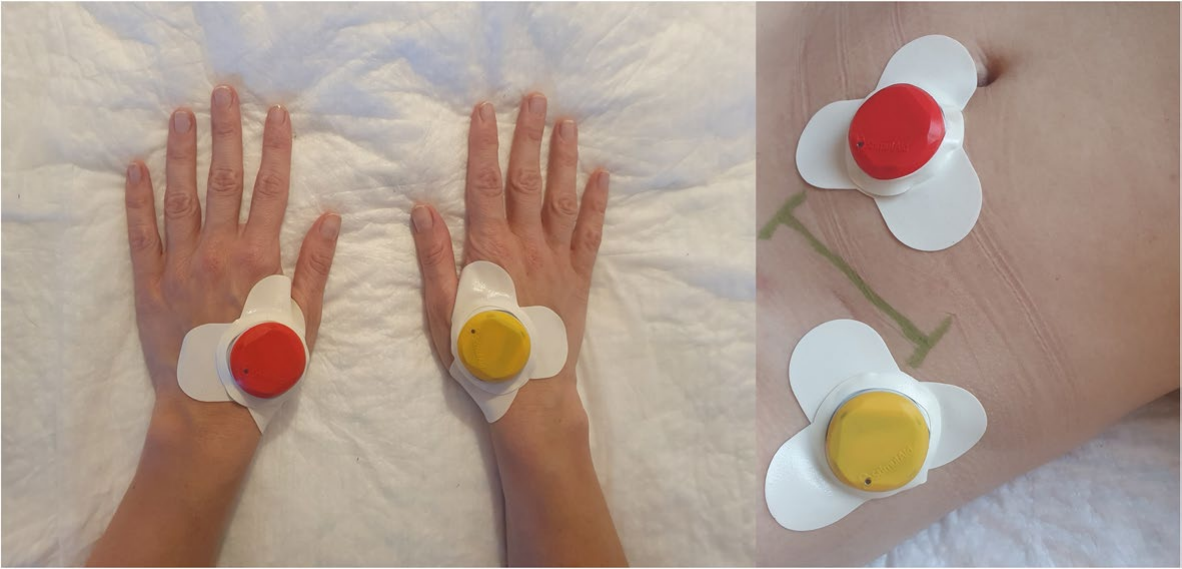
Acupuncture points of treatment. The stimulators will be placed bilaterally to LI4 (left) and ipsilaterally to hernia to two ashi points located within a diameter of 5 cm from the incision site

Stimulation will be performed using four portable coin-sized electro-stimulators (StimulAid Inc, Poland). A point-detection function in the device will confirm the correct localization of the stimulator. A combination of the LI4 acupuncture point and peri-incisional ashi points will be utilized to augment PCA analgesia in the postoperative period [16–18]. Not only point selection but also the frequency of stimulation and treatment timing play a critical role in determining the outcome of the intervention. Low-frequency (2 Hz) electrostimulation applied at the acupoints exerts antinociceptive effects mainly by enhancing the release of met-enkephalin (M-ENK), β-endorphin (β-EP), and dynorphin [19, 20]. Therefore, the TEAS group will receive mixed frequency stimulation (alternating at 2 and 100 Hz every 3 s) in continuous mode for 30 min at intervals of 2 h [19, 20]. The device will automatically shut off at the end of each 30-min treatment interval. The intensity of the stimulation will be adjusted to each individual to maintain a slight twitching of the regional muscle and achieve De-Qi sensations, such as soreness, distention, and heaviness. In both groups, electro-stimulators will be applied bilaterally to LI4 (He Gu) and ipsilateral to hernia to two ashi points located within a diameter of 5 cm from the incision site (Fig. 3). The sham group will be provided with the same devices as TEAS with the “in use” light flashing in the usual manner; however, the participants will be told that they may not be able to feel the electrical stimulation. The patients in TEAS and sham groups will be told that they are receiving current stimulation. The PCA therapy and TEAS/sham will be discontinued at 24 h.

## Adverse events

All adverse events will be closely monitored through reports by participants or direct observation by personnel and by asking the patients about adverse events during the observation period. All adverse reactions will be recorded, and additional treatment will be offered if required. Serious adverse events will be reported to the ethics committee.

## Post-trial care

After the conclusion of a trial, the follow-up care will be provided by the patient’s personal physician.

## Data management

Demographic and clinical data will be gathered from all participants. All adverse events will be documented. Data will be collected by a surgical nurse and securely managed by the study coordinator. The data will be available only to investigators and to institutional or governmental auditors. The scientific research management committee will have access to the final trial dataset.

## Data monitoring and dissemination policy

Data and Safety Monitoring Board will evaluate the trial data periodically for integrity and to assure the safety of clinical trial participants. At the end of the study, the original data and results will be submitted to the scientific research management committee; they will be disclosed to the public after the results are published.

## Protocol amendments

If the protocol changes during the implementation of the study, researchers will communicate the important protocol modifications (e.g., changes to eligibility criteria, outcomes, analyses) to the relevant parties (e.g., investigators, REC/IRBs, trial participants, trial registries, journals, regulators).

## Discussion

The study aims to evaluate the efficacy and safety of TEAS for postoperative pain after inguinal hernia repair compared with sham and standard treatment. When planning a TEAS study, treatment timing, acupuncture points, and frequency of stimulation play a critical role in the outcome of the intervention. In several TENS and EA trials for postoperative pain, the number of stimulation sessions was limited to 4 or 5. It is likely that an intensified treatment protocol, which consists of multiple 30-min sessions at intervals of 2 h, will produce a more significant effect. To the best of our knowledge, this is the first study to examine an intensified stimulation protocol with TEAS in reducing analgesic consumption after surgery.

A mounting body of evidence suggests that acupuncture and acupuncture-related techniques can be performed before, during, or after surgery to alleviate postoperative pain [21]. TEAS or acupuncture-like TENS become more acceptable than acupuncture given its non-invasive character and similar analgesic effect [22, 23]. Also, the modality has several other advantages, such as programmable, fully automatic stimulation, ease of use, comfort and convenience for the patient, and minimal effort of medical personnel.

However, there is still scarce data to document the effects of TEAS on surgical pain [24, 25]. Adding the fact that it is easy to employ TEAS in a postoperative setting and that multiple stimulation sessions are possible, we planned to perform the trial to assess whether TEAS could be more effective in reducing the opioid requirement and improving patient comfort after surgery.

To maximize the effect of acupuncture treatment, we utilized both distal and local points. Several trials have used only distal points, including LI4 and ST36 [16–18], or only local points around the incision region [15]; however, a combination of points prove to exert a strong analgesic effect [14, 26]. Many clinicians assume that direct electrical stimulation around the incision region may lead to adverse events, but to date, there has not been any evidence of harmful effects such as increased pain or infection.

The limitation of our trial is that the assessors/medical personnel is not blinded. Due to the limits of the existing technology, we are unable to blind medical personnel. However, in the future, we may be able to utilize identical pre-programmed devices for sham and real group to resolve the blinding issue. Moreover, this trial is a single-center study. As the curative effect of TEAS may be affected by ethnicity and region, it will be essential to perform multicenter and large sample experiments in the future.

We anticipate that the results will determine whether TEAS with intensified stimulation protocol is a safe and effective option for reducing analgesic consumption and postoperative pain. The results of the study may serve as a platform for developing TEAS-based treatment regimens for enhanced recovery after surgery.

## Trial status

The study registration number is KB 599/2017 dated 21/07/2017. The first participant was enrolled on 01/01/2019. Recruitment was completed on 31/12/2020.

## Data Availability

The authors confirm that the data supporting the findings of this study are available within the article. The results will be disseminated in peer-reviewed journals and presented at international conferences.
